# Current Awareness on Comparative and Functional Genomics

**DOI:** 10.1002/cfg.420

**Published:** 2005-04


**Comparative and Functional Genomics**
*Comp Funct Genom* 2005; **6**: 185–192
Published online in Wiley InterScience (www.interscience.wiley.com). **DOI:** 10.1002cfg.420

### Current awareness on comparative and functional genomics

CURRENT AWARENESS - USE THE LINKS ONLINE

View this bibliography online at WILEY InterScience www.interscience.wiley.com

Your gateway to Wiley journals online

The articles use crossref, the innovative multi-publisher reference linking. This enables readers to move seamlessly from the reference to the cited publication, typically located on a different server and published by a different publisher. Links to MEDLINE abstracts are also available for key journals in the biosciences.

2745

### 1 Reviews & symposia

Aigner T, Bartnik E, Sohler F, Zimmer R. 2004. Functional genomics of osteoarthritis - On the way to evaluate disease hypotheses. *Clin Orthop* **427:** (Suppl) S138.

Archer DB, Dyer PS. 2004. From genomics to post-genomics in *Aspergillus*. *Curr Opin Microbiol* **7:** (5) 499.

Bino R, Hall R, Fiehn O, Kopka J, Saito K, Draper J, Nikolau B, Mendes P, Roessner-Tunali U, Beale MH *et al*. 2004. Potential of metabolomics as a functional genomics tool. *Trends Plant Sci* **9:** (9) 418.

Borevitz JO, Ecker JR. 2004. Plant genomics: The third wave. *Annu Rev Genomic Hum Genet* **5:** 443.

Brazeau DA. 2004. Combining genome-wide and targeted gene expression profiling in drug discovery: Microarrays and real-time PCR (Review). *Drug Discov Today* **9:** (19) 838.

Bullerwell CE, Gray MW. 2004. Evolution of the mitochondrial genome: Protist connections to animals, fungi and plants. *Curr Opin Microbiol* **7:** (5) 528.

Bumann D, Jungblut PR, Meyer TF. 2004. *Helicobacter pylori* vaccine development based on combined subproteome analysis (Review). *Proteomics* **4:** (10) 2843.

Cascorbi L, Paul M, Kroemer HK. 2004. Pharmacogenomics of heart failure - Focus on drug disposition and action (Review). *Cardiovasc Res* **64:** (1) 32.

Chaurand P, Sanders ME, Jensen RA, Caprioli RM. 2004. Proteomics in diagnostic pathology - Profiling and imaging proteins directly in tissue sections (Review). *Am J Pathol* **165:** (4) 1057.

De Hoog CL, Mann M. 2004. Proteomics. *Annu Rev Genomic Hum Genet* **5:** 267.

Devauchelle V, Chiocchia G. 2004. What place for DNA microarray in inflammatory disease? (Review) (French, English Abstract). *Rev Med Interne* **25:** (10) 732.

Enard W, Paabo S. 2004. Comparative primate genomics. *Annu Rev Genomic Hum Genet* **5:** 351.

Fountoulakis M. 2004. Application of proteomics technologies in the investigation of the brain. *Mass Spectrom Rev* **23:** (4) 231.

Freiberg C, Brotz-Oesterhelt H, Labischinski H. 2004. The impact of transcriptome and proteome analyses on antibiotic drug discovery. *Curr Opin Microbiol* **7:** (5) 451.

Fulop AK, Falus A. 2004. Possibilities and results in the wide-scale genomic analysis of inflammation - Looking for the child among many midwives (Review). *Inflamm Res* **53:** (10) 517.

Gil R, Latorre A, Moya A. 2004. Bacterial endosymbionts of insects: Insights from comparative genomics (Review). *Environ Microbiol* **6:** (11) 1109.

Harada K, Xia ZJ. 2004. Soybean genomics: Efforts to reveal the complex genome (Review). *Breeding Sci* **54:** (3) 215.

Hermann T. 2004. Using functional genomics to improve productivity in the manufacture of industrial biochemicals. *Curr Opin Biotechnol* **15:** (5) 444.

Hughes TR, Robinson MD, Mitsakakis N, Johnston M. 2004. The promise of functional genomics: Completing the encyclopedia of a cell. *Curr Opin Microbiol* **7:** (5) 546.

Jansen R, Gerstein M. 2004. Analyzing protein function on a genomic scale: The importance of gold-standard positives and negatives for network prediction. *Curr Opin Microbiol* **7:** (5) 535.

Knaust A, Frosch M. 2004. Genome-based vaccines (Review). *Int J Med Microbiol* **294:** (5) 295.

Kunz M, Ibrahim SM, Koczan D, Scheid S, Thiesen HJ, Gross G. 2004. DNA microarray technology and its applications in dermatology (Review). *Exp Dermatol* **13:** (10) 593.

Maguire PB, Moran N, Cagney G, Fitzgerald DJ. 2004. Application of proteomics to the study of platelet regulatory mechanisms. *Trends Cardiovasc Med* **14:** (6) 207.

Mann KG, Brummel-Ziedins K, Undas A, Butenas S. 2004. Does the genotype predict the phenotype? Evaluations of the hemostatic proteome (Review). *J Thromb Haemost* **2:** (10) 1722.

Marko-Varga G. 2004. Proteomics principles and challenges. *Pure Appl Chem* **76:** (4) 829.

Meschia JF. 2004. Clinically translated ischemic stroke genomics. *Stroke* **35:** (11 Suppl 1) 2735.

Miller W, Makova KD, Nekrutenko A, Hardison RC. 2004. Comparative genomics. *Annu Rev Genomic Hum Genet* **5:** 15.

Minagar A, Shapshak P, Duran EM, Kablinger AS, Alexander JS, Kelley RE, Seth R, Kazic T. 2004. HIV-associated dementia, Alzheimer's disease, multiple sclerosis, and schizophrenia: Gene expression review (Review). *J Neurol Sci* **224:** (1-2) 3.

Mischel PS, Cloughesy TF, Nelson SF. 2004. DNA-microarray analysis of brain cancer: Molecular classification for therapy. *Nat Rev Neurosci* **5:** (10) 782.

Misteli T. 2004. Spatial positioning: A new dimension in genome function (Review). *Cell* **119:** (2) 153.

Moreira LM, De Souza RE, Almeida NF, Setubal JC, Oliveira JCF, Furlan LR, Ferro JA, Da Silva ACR. 2004. Comparative genomics

In order to keep subscribers up-to-date with the latest developments in their field, this current awareness service is provided by John Wiley & Sons and contains newly-published material on comparative and functional genomics. Each bibliography is divided into 16 sections. 1 Reviews & symposia; 2 General; 3 Large-scale sequencing and mapping; 4 Evolutionary genomics; 5 Comparative genomics; 6 Pathways, gene families and regulons; 7 Pharmacogenomics; 8 EST, cDNA and other clone resources; 9 Functional genomics; 10 Transcriptomics; 11 Proteomics; 12 Protein structural genomics; 13 Metabolomics; 14 Genomic approaches to development; 15 Technological advances; 16 Bioinformatics. Within each section, articles are listed in alphabetical order with respect to author. If, in the preceding period, no publications are located relevant to any one of these headings, that section will be omitted.

Copyright © 2005 John Wiley & Sons, Ltd.

analyses of citrus-associated bacteria. *Annu Rev Phytopathol* **42:** 163.
Noirot P, Noirot-Gros MF. 2004. Protein interaction networks in bacteria. *Curr Opin Microbiol* **7:** (5) 505.
Ordovas JM, Corella D. 2004. Nutritional genomics. *Annu Rev Genomic Hum Genet* **5:** 71.
Parsons L, Liu F, Yeh DC, Sari N, Orban J. 2004. Integrating structural and functional genomics. *Aust J Chem* **57:** (7) 619.
Pasa-Tolic L, Masselon C, Barry RC, Shen YF, Smith RD. 2004. Proteomic analyses using an accurate mass and time tag strategy (Review). *Biotechniques* **37:** (4) 621.
Petros WP, Evans WE. 2004. Pharmacogenomics in cancer therapy: Is host genome variability important? (Review). *Trends Pharmacol Sci* **25:** (9) 457.
Righetti PG, Castagna A, Antonucci F, Piubelli C, Cecconi D, Campostrini N, Antonioli P, Astner H, Hamdan M. 2004. Critical survey of quantitative proteomics in two-dimensional electrophoretic approaches. *J Chromatogr A* **1051:** (1-2) 3.
Rocha EPC. 2004. Order and disorder in bacterial genomes. *Curr Opin Microbiol* **7:** (5) 519.
Sagoo GS, Cork MJ, Patel R, Tazi-Ahnini R. 2004. Genome-wide studies of psoriasis susceptibility loci: A review. *J Dermatol Sci* **35:** (3) 171.
Serruto D, Adu-Bobie J, Capecchi B, Rappuoli R, Pizza M, Masignani V. 2004. Biotechnology and vaccines: Application of functional genomics to *Neisseria meningitidis* and other bacterial pathogens. *J Biotechnol* **113:** (1-3) 15.
Sharp RE, Poroyko V, Hejlek LG, Spollen WG, Springer GK, Bohnert HJ, Nguyen HT. 2004. Root growth maintenance during water deficits: Physiology to functional genomics. *J Exp Bot* **55:** (407) 2343.
Shi Y, Xiang R, Horvath C, Wilkins JA. 2004. The role of liquid chromatography in proteomics (Review). *J Chromatogr A* **1053:** (1-2) 27.
Soltis DE, Albert VA, Savolainen V, Hilu K, Qiu YL, Chase MW, Farris JS, Stefanovic S, Rice DW, Palmer JD *et al*. 2004. Genome-scale data, angiosperm relationships, and 'ending incongruence': A cautionary tale in phylogenetics. *Trends Plant Sci* **9:** (10) 477.
Stelling J. 2004. Mathematical models in microbial systems biology. *Curr Opin Microbiol* **7:** (5) 513.
Streit WR, Schmitz RA. 2004. Metagenomics - The key to the uncultured microbes. *Curr Opin Microbiol* **7:** (5) 492.
Uetz P, Rajagopala SV, Dong YA, Haas J. 2004. From ORFeomes to protein interaction maps in viruses (Review). *Genome Res* **14:** (10B) 2029.
Vandahl BBS, Birkelund S, Christiansen G. 2004. Genome and proteome analysis of *Chlamydia* (Review). *Proteomics* **4:** (10) 2831.
Veenstra TD, Prieto DA, Conrads TP. 2004. Proteomic patterns for early cancer detection. *Drug Discov Today* **9:** (20) 889.
Vercauteren FGG, Bergeron JJM, Vandesande F, Arckens L, Quirion R. 2004. Proteomic approaches in brain research and neuropharmacology. *Eur J Pharmacol* **500:** (1-3) 385.
Wang XD, Zhao H, Andersson R. 2004. Proteomics and leukocytes: An approach to understanding potential molecular mechanisms of inflammatory responses (Review). *J Proteome Res* **3:** (5) 921.
Warnock DE, Fahy E, Taylor SW. 2004. Identification of protein associations in organelles, using mass spectrometry-based proteomics. *Mass Spectrom Rev* **23:** (4) 259.
Yokoyama R, Nishitani K. 2004. Genomic basis for cell-wall diversity in plants: A comparative approach to gene families in rice and *Arabidopsis* (Review). *Plant Cell Physiol* **45:** (9) 1111.

### 3 Large-scale sequencing and mapping

Intl Hum Genome Seq Consortium. 2004. Finishing the euchromatic sequence of the human genome. *Nature* **431:** (7011) 931.
Grayburn WS, Hudspeth DSS, Gane MK, Hudspeth MES. 2004. The mitochondrial genome of *Saprolegnia ferax*: Organization, gene content and nucleotide sequence. *Mycologia* **96:** (5) 981.
Hong S, Kim J, Lee S, In Y, Choi S, Rih J, Kim CH, Jeong H, Hur CG, Kim JJ. 2004. The genome sequence of the capnophilic rumen bacterium *Mannheimia succiniciproducens*. *Nat Biotechnol* **22:** (10) 1275.
Ishikawa J, Yamashita A, Mikami Y, Hoshino Y, Kurita H, Hotta K, Shiba T, Hattori M. 2004. The complete genomic sequence of *Nocardia farcinica* IFM 10152. *Proc Natl Acad Sci U S A* **101:** (41) 14925.
Nosek J, Novotna M, Hlavatovicova Z, Ussery DW, Fajkus J, Tomaska L. 2004. Complete DNA sequence of the linear mitochondrial genome of the pathogenic yeast *Candida parapsilosis*. *Mol Genet Genomics* **272:** (2) 173.
She XW, Jiang ZX, Clark RL, Liu G, Cheng Z, Tuzun E, Church DM, Sutton G, Halpern AL, Eichler EE. 2004. Shotgun sequence assembly and recent segmental duplications within the human genome. *Nature* **431:** (7011) 927.
Ueda K, Yamashita A, Ishikawa J, Shimada M, Watsuji T, Morimura K, Ikeda H, Hattori M, Beppu T. 2004. Genome sequence of *Symbiobacterium thermophilum*, an uncultivable bacterium that depends on microbial commensalism. *Nucleic Acids Res* **32:** (16) 4937.
Whitton C, Daub J, Quail M, Hall N, Foster J, Ware J, Ganatra M, Slatko B, Barrell B, Blaxter M. 2004. A genome sequence survey of the filarial nematode *Brugia malayi*: Repeats, gene discovery, and comparative genomics. *Mol Biochem Parasitol* **137:** (2) 215.
Xu P, Widmer G, Wang YP, Ozaki LS, Alves JM, Serrano MG, Puiu D, Manque P, Akiyoshi D, Mackey AJ *et al*. 2004. The genome of *Cryptosporidium hominis*. *Nature* **431:** (7012) 1107.

### 4 Evolutionary genomics

Babenko VN, Krylov DM. 2004. Comparative analysis of complete genomes reveals gene loss, acquisition and acceleration of evolutionary rates in Metazoa, suggests a prevalence of evolution *via* gene acquisition and indicates that the evolutionary rates in animals tend to be conserved. *Nucleic Acids Res* **32:** (17) 5029.
Chain PSG, Carniel E, Larimer FW, Lamerdin J, Stoutland PO, Regala WM, Georgescu AM, Vergez LM, Land ML, Motin VL *et al*. 2004. Insights into the evolution of *Yersinia pestis* through whole-genome comparison with *Yersinia pseudotuberculosis*. *Proc Natl Acad Sci U S A* **101:** (38) 13826.
Danchin EGJ, Pontarotti P. 2004. Statistical evidence for a more than 800-million-year-old evolutionarily conserved genomic region in our genome. *J Mol Evol* **59:** (5) 587.
Eberl L, Tummler B. 2004. *Pseudomonas aeruginosa* and *Burkholderia cepacia* in cystic fibrosis: Genome evolution, interactions and adaptation. *Int J Med Microbiol* **294:** (2-3) 123.
Espagne E, Dupuy C, Huguet E, Cattolico L, Provost B, Martins N, Poirie M, Periquet G, Drezen JM. 2004. Genome sequence of a polydnavirus: Insights into symbiotic virus evolution. *Science* **306:** (5694) 286.
Hagopian JC, Reis M, Kitajima JP, Bhattacharya D, De Oliveira MC. 2004. Comparative analysis of the complete plastid genome sequence of the red alga *Gracilaria tenuistipitata* var. *liui* provides insights into the evolution of rhodoplasts and their relationship to other plastids. *J Mol Evol* **59:** (4) 464.
Jaillon O, Aury JM, Brunet F, Petit JL, Stange-Thomann N, Mauceli E, Bouneau L, Fischer C, Ozouf-Costaz C, Bernot A *et al*. 2004. Genome duplication in the teleost fish *Tetraodon nigroviridis* reveals the early vertebrate protokaryotype. *Nature* **431:** (7011) 946.
Macey JR, Papenfuss TJ, Kuehl JV, Fourcade HM, Boore JL. 2004. Phylogenetic relationships among amphisbaenian reptiles based on complete mitochondrial genomic sequences. *Mol Phylogenet Evol* **33:** (1) 22.
Mueller RL, Macey JR, Jaekel M, Wake DB, Boore JL. 2004. Morphological homoplasy, life history evolution, and historical biogeography of plethodontid salamanders inferred from complete mitochondrial genomes. *Proc Natl Acad Sci U S A* **101:** (38) 13820.
Novichkov PS, Omelchenko MV, Gelfand MS, Mironov AA, Wolf YI, Koonin EV. 2004. Genome-wide molecular clock and horizontal gene transfer in bacterial evolution. *J Bacteriol* **186:** (19) 6575.
Pearson T, Busch JD, Ravel J, Read TD, Rhoton SD, U'Ren JM, Simonson TS, Kachur SM, Leadem RR, Cardon ML *et al*. 2004. Phylogenetic discovery bias in *Bacillus anthracis* using single-nucleotide polymorphisms from whole-genome sequencing. *Proc Natl Acad Sci U S A* **101:** (37) 13536.
Zhu J, Lum PY, Lamb J, Guha Thakurta D, Edwards SW, Thieringer R, Berger JP, Wu MS, Thompson J, Sachs AB *et al*. 2004. An integrative genomics approach to the reconstruction of gene networks in segregating populations. *Cytogenet Genome Res* **105:** (2-4) 363.

Copyright © 2005 John Wiley & Sons, Ltd.

### 5 Comparative genomics

Caldwell KS, Langridge P, Powell W. 2004. Comparative sequence analysis of the region harboring the hardness locus in barley and its colinear region in rice. *Plant Physiol* **136:** (2) 3177.

Feng Q, Yu H, Liu YY, He CQ, Hu JS, Sang HC, Ding NZ, Ding MX, Fung YWW, Lau LT *et al*. 2004. Genome comparison of a novel foot-and-mouth disease virus with other FMDV strains. *Biochem Biophys Res Commun* **323:** (1) 254.

Qiu DR, Fujita K, Sakuma Y, Tanaka T, Ohashi Y, Ohshima H, Tomita M, Itaya M. 2004. Comparative analysis of physical maps of four *Bacillus subtilis* (natto) genomes. *Appl Environ Microbiol* **70:** (10) 6247.

Taboada EN, Acedillo RR, Carrillo CD, Findlay WA, Medeiros DT, Mkytczuk OL, Roberts MJ, Valencia CA, Farber JM, Nash JHE. 2004. Large-scale comparative genomics meta-analysis of *Campylobacter jejuni* isolates reveals low level of genome plasticity. *J Clin Microbiol* **42:** (10) 4566.

Zhou DS, Han YP, Song YJ, Huang PT, Yang RF. 2004. Comparative and evolutionary genomics of *Yersinia pestis*. *Microbes Infect* **6:** (13) 1226.

### 7 Pharmacogenomics

Alzate O, Hussain SRA, Goett VM, Tewari AK, Madiai F, Stephens RL, Hackshaw KV. 2004. Proteomic identification of brainstem cytosolic proteins in a neuropathic pain model. *Mol Brain Res* **128:** (2) 193.

Becker S, Cazares LH, Watson P, Lynch H, Semmes OJ, Drake RR, Laronga C. 2004. Surfaced-enhanced laser desorption/ionization time-of-flight (SELDI-TOF) differentiation of serum protein profiles of BRCA-1 and sporadic breast cancer. *Ann Surg Oncol* **11:** (10) 907.

Bhattacharyya S, Siegel ER, Petersen GM, Chari ST, Suva LJ, Haun RS. 2004. Diagnosis of pancreatic cancer using serum proteomic profiling. *Neoplasia* **6:** (5) 674.

Bianchi F, Hu JT, Pelosi G, Cirincione R, Ferguson M, Ratcliffe C, Di Fiore PP, Gatter K, Pezzella F, Pastorino U. 2004. Lung cancers detected by screening with spiral computed tomography have a malignant phenotype when analyzed by cDNA microarray. *Clin Cancer Res* **10:** (18) 6023.

Bibikova M, Talantov D, Chudin E, Yeakley JM, Chen J, Doucet D, Wickham E, Atkins D, Barker D, Chee M *et al*. 2004. Quantitative gene expression profiling in formalin-fixed, paraffin-embedded tissues using universal bead arrays. *Am J Pathol* **165:** (5) 1799.

Bieche I, Lerebours F, Tozlu S, Espie M, Marty M, Lidereau R. 2004. Molecular profiling of inflammatory breast cancer: Identification of a poor-prognosis gene expression signature. *Clin Cancer Res* **10:** (20) 6789.

Boots AM, Verhaert PD, Poele RJT, Evers S, Coenen-de Roo CJ, Cleven J, Bos ES. 2004. Antigens up the nose: Identification of putative biomarkers for nasal tolerance induction functional studies combined with proteomics. *J Proteome Res* **3:** (5) 1056.

Brasaemle DL, Dolios G, Shapiro L, Wang R. 2004. Proteomic analysis of proteins associated with lipid droplets of basal and lipolytically stimulated 3T3-L1 adipocytes. *J Biol Chem* **279:** (45) 46835.

Brennan JP, Wait R, Begum S, Bell JR, Dunn MJ, Eaton P. 2004. Detection and mapping of widespread intermolecular protein disulfide formation during cardiac oxidative stress using proteomics with diagonal electrophoresis. *J Biol Chem* **279:** (40) 41352.

Breuza L, Halbeisen R, Jeno P, Otte S, Barlowe C, Hong WJ, Hauri HP. 2004. Proteomics of endoplasmic reticulum-Golgi intermediate compartment (ERGIC) membranes from brefeldin A-treated HepG2 cells identifies ERGIC-32, a new cycling protein that interacts with human Erv46. *J Biol Chem* **279:** (45) 47242.

Brunori L, Giannoni F, Bini L, Liberatori S, Frota C, Jenner P, Thoresen OF, Orefici G, Fattorini L. 2004. Induction of *Mycobacterium avium* proteins upon infection of human macrophages. *Proteomics* **4:** (10) 3078.

Cao QJ, Belbin T, Socci N, Balan R, Prystowsky MB, Childs G, Jones JG. 2004. Distinctive gene expression profiles by cDNA microarrays in endometrioid and serous carcinomas of the endometrium. *Int J Gynecol Pathol* **23:** (4) 321.

Castagna A, Antonioli P, Astner H, Hamdan M, Righetti SC, Perego P, Zunino F, Righetti PG. 2004. A proteomic approach to cisplatin resistance in the cervix squamous cell carcinoma cell line A431. *Proteomics* **4:** (10) 3246.

Chen J, Kahne T, Rocken C, Gotze T, Yu J, Sung JJY, Chen MH, Hu PJ, Malfertheiner P, Ebert MPA. 2004. Proteome analysis of gastric cancer metastasis by two-dimensional gel electrophoresis and matrix assisted laser desorption/ionization-mass spectrometry for identification of metastasis-related proteins. *J Proteome Res* **3:** (5) 1009.

Chen YJ, Lin SC, Kao T, Chang CS, Hong PS, Shieh TM, Chang KW. 2004. Genome-wide profiling of oral squamous cell carcinoma. *J Pathol* **204:** (3) 326.

Chen YJ, Shi GY, Wei X, Kong C, Zhao SC, Gaw AF, Chen EY, Yang GP, Giaccia AJ, Le QT *et al*. 2004. Identification of hypoxia-regulated proteins in head and neck cancer by proteomic and tissue array profiling. *Cancer Res* **64:** (20) 7302.

Chen ZJ, Peng B, Wang SY, Peng XX. 2004. Rapid screening of highly efficient vaccine candidates by immunoproteomics. *Proteomics* **4:** (10) 3203.

Christopher K, Liang YR, Mueller TF, DeFina R, He HZ, Haley KJ, Exley MA, Finn PW, Perkins DL. 2004. Analysis of the major histocompatibility complex in graft rejection revisited by gene expression profiles. *Transplantation* **78:** (6) 788.

Cui JF, Liu YK, Pan BS, Song HY, Zhang Y, Sun RX, Chen J, Feng JT, Tang ZY, Yu YL *et al*. 2004. Differential proteomic analysis of human hepatocellular carcinoma cell line metastasis-associated proteins. *J Cancer Res Clin Oncol* **130:** (10) 615.

Cui JW, Wang J, He K, Jin BF, Wang HX, Li W, Kang LH, Hu MR, Li HY, Yu M *et al*. 2004. Proteomic analysis of human acute leukemia cells: Insight into their classification. *Clin Cancer Res* **10:** (20) 6887.

De Cecco L, Marchionni L, Gariboldi M, Reid JF, Lagonigro MS, Caramuta S, Ferrario C, Bussani E, Mezzanzanica D, Turatti F *et al*. 2004. Gene expression profiling of advanced ovarian cancer: Characterization of a molecular signature involving fibroblast growth factor 2. *Oncogene* **23:** (49) 8171.

Donninger H, Bonome T, Radonovich M, Pise-Masison CA, Brady J, Shih JH, Barrett JC, Birrer MJ. 2004. Whole genome expression profiling of advance stage papillary serous ovarian cancer reveals activated pathways. *Oncogene* **23:** (49) 8065.

Dvorzhinski D, Thalasila A, Thomas PE, Nelson D, Li H, White E, DiPaola RS. 2004. A novel proteomic coculture model of prostate cancer cell growth. *Proteomics* **4:** (10) 3268.

Engellau J, Persson A, Bendahl PO, Akerman M, Domanski HA, Bjerkehagen B, Lilleng P, Weide J, Rydhohn A, Alvegard TA *et al*. 2004. Expression profiling using tissue microarray in 211 malignant fibrous histiocytomas confirms the prognostic value of Ki-67. *Virchows Archiv* **445:** (3) 224.

Feng J, Yin ZN, Caraway NP, Li RY, Katz RL. 2004. Genomic profiles in stage I primary non small cell lung cancer using comparative genomic hybridization analysis of cDNA microarrays. *Neoplasia* **6:** (5) 623.

Fernandez-Arenas E, Molero G, Nombela C, Diez-Orejas R, Gil C. 2004. Low virulent strains of *Candida albicans*: Unravelling the antigens for a future vaccine. *Proteomics* **4:** (10) 3007.

Franscini N, Bachli EB, Blau N, Leikauf MS, Schaffner A, Schoedon G. 2004. Gene expression profiling of inflamed human endothelial cells and influence of activated protein C. *Circulation* **110:** (18) 2903.

Gao XQ, Han JX, Huang HY, Yan S, Song CZ, Huang HN. 2004. Effects of aspirin on metastasis-associated gene expression detected by cDNA microarray. *Acta Pharmacol Sin* **25:** (10) 1327.

Gehrmann ML, Hathout Y, Fenselau C. 2004. Evaluation of metabolic labeling for comparative proteomics in breast cancer cells. *J Proteome Res* **3:** (5) 1063.

Greenough C, Jenkins RE, Kitteringham NR, Pirmohamed M, Park BK, Pennington SR. 2004. A method for the rapid depletion of albumin and immunoglobulin from human plasma. *Proteomics* **4:** (10) 3107.

Gronborg M, Bunkenborg J, Kristiansen TZ, Jensen ON, Yeo CJ, Hruban RH, Maitra A, Goggins MG, Pandey A. 2004. Comprehensive proteomic analysis of human pancreatic juice. *J Proteome Res* **3:** (5) 1042.

Grutzmann R, Pilarsky C, Ammerpohl O, Luttges J, Bohme A, Sipos B, Foerder M, Alldinger I, Jahnke B, Schackert HK *et al*. 2004. Gene expression profiling of microdissected pancreatic ductal carcinomas using high-density DNA microarrays. *Neoplasia* **6:** (5) 611.

Harris J, Mason DE, Li J, Burdick KW, Backes BJ, Chen T, Shipway A,

Copyright © 2005 John Wiley & Sons, Ltd. 

Van Heeke G, Gough L, Ghaemmaghami A *et al*. 2004. Activity profile of dust mite allergen extract using substrate libraries and functional proteomic microarrays. *Chem Biol* **11:** (10) 1361.

Haslinger C, Schweifer N, Stilgenbauer S, Dohner H, Lichter P, Kraut N, Stratowa C, Abseher R. 2004. Microarray gene expression profiling of B-cell chronic lymphocytic leukemia subgroups defined by genomic aberrations and VH mutation status. *J Clin Oncol* **22:** (19) 3937.

Hata F, Nishimori H, Yasoshima T, Tanaka H, Ohno K, Yanai Y, Ezoe E, Kamiguchi K, Isomura H, Denno R *et al*. 2004. Profiling analysis of differential gene expression between hematogenous and peritoneal metastatic sublines of human pancreatic cancer using a DNA chip. *J Exp Clin Cancer Res* **23:** (3) 513.

He QY, Cheung YH, Leung SY, Yuen ST, Chu KM, Chiu JF. 2004. Diverse proteomic alterations in gastric adenocarcinoma. *Proteomics* **4:** (10) 3276.

Hermansson M, Sawaji Y, Bolton M, Alexander S, Wallace A, Begum S, Wait R, Saklatvala J. 2004. Proteomic analysis of articular cartilage shows increased type II collagen synthesis in osteoarthritis and expression of inhibin βA (activin A), a regulatory molecule for chondrocytes. *J Biol Chem* **279:** (42) 43514.

Hivnor C, Williams N, Singh F, Van Voorhees A, Dzubow L, Baldwin D, Seykora J. 2004. Gene expression profiling of porokeratosis demonstrates similarities with psoriasis. *J Cutan Pathol* **31:** (10) 657.

Huang ZY, Yang PY, Almofti MR, Yu YL, Rui YC, Yang PY. 2004. Comparative analysis of the proteome of left ventricular heart of arteriosclerosis in rat. *Life Sci* **75:** (26) 3103.

Hubalek M, Hernychova L, Brychta M, Lenco J, Zechovska J, Stulik J. 2004. Comparative proteome analysis of cellular proteins extracted from highly virulent *Francisella tularensis* ssp *tularensis* and less virulent *F. tularensis* ssp *holarctica* and *F. tularensis* ssp *mediaasiatica*. *Proteomics* **4:** (10) 3048.

Hurst AGB, Goad DW, Mohan M, Malayer JR. 2004. Independent downstream gene expression profiles in the presence of estrogen receptor α or β. *Biol Reprod* **71:** (4) 1252.

Izumi Y, Izumiya Y, Shiota M, Yukimura T, Shiojima S, Yamada M, Kadowaki T, Katsuma S, Hirasawa A, Tsujimoto G *et al*. 2004. Gene expression profile in experimental mesangial proliferative glomerulonephritis. *J Pharmacol Sci* **96:** (1) 91.

Jeon GA, Lee JS, Patel V, Gutkind JS, Thorgeirsson SS, Kim FC, Chu IS, Amornphimoltham P, Park MH. 2004. Global gene expression profiles of human head and neck squamous carcinoma cell lines. *Int J Cancer* **112:** (2) 249.

Ju XS, Zenke M. 2004. Gene expression profiling of dendritic cells by DNA microarrays. *Immunobiology* **209:** (1-2) 155.

Kainuma K, Katsuno S, Hashimoto S, Suzuki N, Oguchi T, Asamura K, Nakajima K, Usami S. 2004. Identification of differentially expressed genes in salivary gland tumors with cDNA microarray. *Auris Nasus Larynx* **31:** (3) 261.

Kan B, Habibi H, Schmid M, Liang W, Wang RB, Wang DC, Jungblut PR. 2004. Proteome comparison of *Vibrio cholerae* cultured in aerobic and anaerobic conditions. *Proteomics* **4:** (10) 3061.

Kavsan V, Shostak K, Dmitrenko V, Chausovskiy T, Zozulya Y, Demotes-Mainard J. 2004. Peculiarities of molecular events in human glial tumors revealed by serial analysis of gene expression (SAGE). *Exp Oncol* **26:** (3) 196.

Komori T, Takemasa I, Higuchi H, Yamasaki M, Ikeda M, Yamamoto H, Ohue M, Nakamori S, Sekimoto M, Matsubara K *et al*. 2004. Identification of differentially expressed genes involved in colorectal carcinogenesis using a cDNA microarray. *J Exp Clin Cancer Res* **23:** (3) 521.

Kwak JY, Ma TZ, Yoo MJ, Choi BH, Kim HG, Kim SR, Yim CY, Kwak YG. 2004. The comparative analysis of serum proteomes for the discovery of biomarkers for acute myeloid leukemia. *Exp Hematol* **32:** (9) 836.

Lau ATY, He QY, Chiu JF. 2004. A proteome analysis of the arsenite response in cultured lung cells: Evidence for *in vitro* oxidative stress-induced apoptosis. *Biochem J* **382:** (2) 641.

Lee HS, Park MH, Yang SJ, Jung HY, Byun SS, Lee DS, Yoo HS, Yeom Y, Seo SB. 2004. Gene expression analysis in human gastric cancer cell line treated with trichostatin A and *S*-adenosyl-L-homocysteine using cDNA microarray. *Biol Pharm Bull* **27:** (10) 1497.

Li LH, Tang H, Wu ZB, Gong JL, Gruidl M, Zou J, Tockman M, Clark RA. 2004. Data mining techniques for cancer detection using serum proteomic profiling. *Artif Intell Med* **32:** (2) 71.

Liu XH, Qian LJ, Gong JB, Shen J, Zhang XM, Qian XH. 2004. Proteomic analysis of mitochondrial proteins in cardiomyocytes from chronic stressed rat. *Proteomics* **4:** (10) 3167.

Mahlamaki EH, Kauraniemi P, Monni O, Wolf M, Hautaniemi S, Kallioniemi A. 2004. High-resolution genomic and expression profiling reveals 105 putative amplification target genes in pancreatic cancer. *Neoplasia* **6:** (5) 432.

Marko-Varga GA, Fehniger TE. 2004. Microscale protein expression profiling during disease evolvement. *J Chromatogr A* **1053:** (1-2) 279.

Mateos-Caceres PJ, Garcia-Mendez A, Farre AL, Macaya C, Nunez A, Gomez J, Alonso-Orgaz S, Carrasco C, Burgos ME, De Andres R *et al*. 2004. Proteomic analysis of plasma from patients during an acute coronary syndrome. *J Am Coll Cardiol* **44:** (8) 1578.

Mehul B, Asselineau D, Bernard D, Leclaire J, Regnier M, Schmidt R, Bernerd F. 2004. Gene expression profiles of three different models of reconstructed human epidermis and classical cultures of keratinocytes using cDNA arrays. *Arch Dermatol Res* **296:** (4) 145.

Mollenkopf HJ, Grode L, Mattow J, Stein M, Mann P, Knapp B, Ulmer J, Kaufmann SHE. 2004. Application of mycobacterial proteomics to vaccine design: Improved protection by *Mycobacterium bovis* BCG prime-Rv3407 DNA boost vaccination against tuberculosis. *Infect Immun* **72:** (11) 6471.

Ning W, Li CJ, Kaminski N, Feghali-Bostwick CA, Alber SM, Di YPP, Otterbein SL, Song RP, Hayashi S, Zhou ZH *et al*. 2004. Comprehensive gene expression profiles reveal pathways related to the pathogenesis of chronic obstructive pulmonary disease. *Proc Natl Acad Sci U S A* **101:** (41) 14895.

Nishidate T, Katagiri T, Lin ML, Mano Y, Miki Y, Kasumi F, Yoshimoto M, Tsunoda T, Hirata K, Nakamura Y. 2004. Genome-wide gene-expression profiles of breast-cancer cells purified with laser microbeam microdissection: Identification of genes associated with progression and metastasis. *Int J Oncol* **25:** (4) 797.

Noh HS, Lee HP, Kim DW, Kang SS, Cho GJ, Rho JM, Choi WS. 2004. A cDNA microarray analysis of gene expression profiles in rat hippocampus following a ketogenic diet. *Mol Brain Res* **129:** (1-2) 80.

Orsetti B, Nugoli M, Cervera N, Lasorsa L, Chuchana P, Ursule L, Nguyen C, Redon R, Du Manoir S, Rodriguez C *et al*. 2004. Genomic and expression profiling of chromosome 17 in breast cancer reveals complex patterns of alterations and novel candidate genes. *Cancer Res* **64:** (18) 6453.

Park J, Yoon S, Kim J, Yeom Y, Kim Y, Kim N. 2004. Identification of novel genes associated with the response to 5-FU treatment in gastric cancer cell lines using a cDNA microarray. *Cancer Lett* **214:** (1) 19.

Pitarch A, Abian J, Carrascal M, Sanchez M, Nombela C, Gil C. 2004. Proteomics-based identification of novel *Candida albicans* antigens for diagnosis of systemic candidiasis in patients with underlying hematological malignancies. *Proteomics* **4:** (10) 3084.

Pogue R, Sebald E, King L, Kronstadt E, Krakow D, Cohn DH. 2004. A transcriptional profile of human fetal cartilage. *Matrix Biol* **23:** (5) 299.

Riemer C, Neidhold S, Burwinkel M, Schwarz A, Schultz J, Kratzschmar J, Monning U, Baier M. 2004. Gene expression profiling of scrapie-infected brain tissue. *Biochem Biophys Res Commun* **323:** (2) 556.

Santin AD, Zhan FH, Bellone S, Palmieri M, Cane S, Bignotti E, Anfossi S, Gokden M, Dunn D, Roman JJ *et al*. 2004. Gene expression profiles in primary ovarian serous papillary tumors and normal ovarian epithelium: Identification of candidate molecular markers for ovarian cancer diagnosis and therapy. *Int J Cancer* **112:** (1) 14.

Shibata H, Nakagawa S, Mayumi T, Tsutsumi Y. 2004. Development of novel drug delivery system (DDS) technologies for proteomic-based drug development. *Biol Pharm Bull* **27:** (10) 1483.

Sironi L, Guerrini U, Tremoli E, Miller I, Gelosa P, Lascialfari A, Zucca I, Eberini I, Gemeiner M, Paoletti R, Gianazza E. 2004. Analysis of pathological events at the onset of brain damage in stroke-prone rats: A proteomics and magnetic resonance imaging approach. *J Neurosci Res* **78:** (1) 115

Snyder JA, Haugen BJ, Buckles EL, Lockatell CV, Johnson DE, Donnenberg MS, Welch RA, Mobley HLT. 2004. Transcriptome of uropathogenic *Escherichia coli* during urinary tract infection. *Infect Immun* **72:** (11) 6373.

Copyright © 2005 John Wiley & Sons, Ltd. 

Sommer AP. 2004. Suffocation of nerve fibers by living nanovesicles: A model simulation - Part II (Letter). *J Proteome Res* **3:** (5) 1086.
Suzuyama K, Shiraishi T, Oishi T, Ueda S, Okamoto H, Furuta M, Mineta T, Tabuchi K. 2004. Combined proteomic approach with SELDI-TOF-MS and peptide mass fingerprinting identified the rapid increase of monomeric transthyretin in rat cerebrospinal fluid after transient focal cerebral ischemia. *Mol Brain Res* **129:** (1-2) 44.
Tsou MT, Ho JY, Lin CH, Chiu JH. 2004. Proteomic analysis finds different myocardial protective mechanisms for median nerve stimulation by electroacupuncture and by local somatothermal stimulation. *Int J Mol Med* **14:** (4) 553.
Tsutsumi C, Ueda M, Miyazaki Y, Yamashita Y, Choi YL, Ota J, Kaneda R, Koinuma K, Fujiwara S, Kisanuki H *et al*. 2004. DNA microarray analysis of dysplastic morphology associated with acute myeloid leukemia. *Exp Hematol* **32:** (9) 828.
Varnum SM, Streblow DN, Monroe ME, Smith P, Auberry KJ, Pasa-Tolic L, Wang D, Camp DG, Rodland K, Wiley S *et al*. 2004. Identification of proteins in human cytomegalovirus (HCMV) particles: The HCMV proteome. *J Virol* **78:** (20) 10960.
Verhoeckx KCM, Bijlsma S, Jespersen S, Ramaker R, Verheij ER, Witkamp RF, Van der Greef J, Rodenburg RJT. 2004. Characterization of anti-inflammatory compounds using transcriptomics, proteomics, and metabolomics in combination with multivariate data analysis. *Int Immunopharmacol* **4:** (12) 1499.
Voisine P, Ruel M, Khan TA, Bianchi C, Xu SH, Kohane I, Libermann TA, Otu H, Saltiel AR, Sellke FW. 2004. Differences in gene expression profiles of diabetic and nondiabetic patients undergoing cardiopulmonary bypass and cardioplegic arrest. *Circulation* **110:** (Suppl 11) II280.
Wang E, Lichtenfels R, Bukur J, Ngalame Y, Panelli MC, Seliger B, Marincola FM. 2004. Ontogeny and oncogenesis balance the transcriptional profile of renal cell cancer. *Cancer Res* **64:** (20) 7279.
Wang YH, Hancock WS, Weber G, Eckerskorn C, Palmer-Toy D. 2004. Free flow electrophoresis coupled with liquid chromatography- mass spectrometry for a proteomic study of the human cell line (K562/CR3). *J Chromatogr A* **1053:** (1-2) 269.
Wehr W, Meyer TF, Jungblut PR, Muller EC, Szczepek AJ. 2004. Action and reaction: *Chlamydophila pneumoniae* proteome alteration in a persistent infection induced by iron deficiency. *Proteomics* **4:** (10) 2969.
Wei JS, Greer BT, Westermann F, Steinberg SM, Son CG, Chen QR, Whiteford CC, Bilke S, Krasnoselsky AL, Cenacchi N *et al*. 2004. Prediction of clinical outcome using gene expression profiling and artificial neural networks for patients with neuroblastoma. *Cancer Res* **64:** (19) 6883.
Xu XQ, Leow CK, Lu X, Zhang XG, Liu JS, Wong WH, Asperger A, Deininger S, Leung HCE. 2004. Molecular classification of liver cirrhosis in a rat model by proteomics and bioinformatics. *Proteomics* **4:** (10) 3235.
Yanagisawa R, Takano H, Inoue K, Ichinose T, Yoshida S, Sadakane K, Takeda K, Yoshino S, Yamaki K, Kumagai Y *et al*. 2004. Complementary DNA microarray analysis in acute lung injury induced by lipopolysaccharide and diesel exhaust particles. *Exp Biol Med* **229:** (10) 1081.
Zhao MM, Chow A, Powers J, Fajardo G, Bernstein D. 2004. Microarray analysis of gene expression after transverse aortic constriction in mice. *Physiol Genomics* **19:** (1) 93.
Zhou XD, Dimachkie MM, Xiong MM, Filemon KT, Arnett FC. 2004. cDNA microarrays reveal distinct gene expression clusters in idiopathic inflammatory myopathies. *Med Sci Monit* **10:** (7) BR191.

### 8 EST, cDNA and other clone resources

Baross A, Butterfield YSN, Coughlin SM, Zeng T, Griffith M, Griffith OL, Petrescu AS, Smailus DE, Khattra J, McDonald HL *et al*. 2004. Systematic recovery and analysis of full-ORF human cDNA clones. *Genome Res* **14:** (10B) 2083.
Dricot A, Rual JF, Lamesch P, Bertin N, Dupuy D, Hao T, Lambert C, Hallez R, Delroisse JM, Vandenhaute J *et al*. 2004. Generation of the *Brucella melitensis* ORFeome version 1.1. *Genome Res* **14:** (10B) 2201.
Hilson P, Allemeersch J, Altmann T, Aubourg S, Avon A, Beynon J, Bhalerao RP, Bitton F, Caboche M, Cannoot B *et al*. 2004. Versatile gene-specific sequence tags for *Arabidopsis* functional genomics: Transcript profiling and reverse genetics applications. *Genome Res* **14:** (10B) 2176.
Lamesch P, Milstein S, Hao T, Rosenberg J, Li N, Sequerra R, Bosak S, Doucette-Stamm L, Vandenhaute J, Hill DE *et al*. 2004. *C. elegans* ORFeome version 3.1: Increasing the coverage of 2064 ORFeome resources with improved gene predictions. *Genome Res* **14:** (10B) 2064.
Rual JF, Ceron J, Koreth J, Hao T, Nicot AS, Hirozane-Kishikawa T, Vandenhaute J, Orkin SH, Hill DE, Van den Heuvel S *et al*. 2004. Toward improving *Caenorhabditis elegans* phenome mapping with an ORFeome-based RNAi library. *Genome Res* **14:** (10B) 2162.
Rual JF, Hirozane-Kishikawa T, Hao T, Bertin N, Li SM, Dricot A, Li N, Rosenberg J, Lamesch P, Vidalain PO *et al*. 2004. Human ORFeome version 1.1: A platform for reverse proteomics. *Genome Res* **14:** (10B) 2128.
Sterky F, Bhalerao RR, Unneberg P, Segerman B, Nilsson P, Brunner AM, Charbonnel-Campaa L, Lindvall JJ, Tandre K, Strauss SH *et al*. 2004. A *Populus* EST resource for plant functional genomics. *Proc Natl Acad Sci U S A* **101:** (38) 13951.
Wu CC, Nimmakayala P, Santos FA, Springman R, Scheuring C, Meksem K, Lightfoot DA, Zhang HB. 2004. Construction and characterization of a soybean bacterial artificial chromosome library and use of multiple complementary libraries for genome physical mapping. *Theor Appl Genet* **109:** (5) 1041.
Yao JB, Ren XN, Ireland JJ, Coussens PM, Smith TPL, Smith GW. 2004. Generation of a bovine oocyte cDNA library and microarray: Resources for identification of genes important for follicular development and early embryogenesis. *Physiol Genomics* **19:** (1) 84.
Zhang HN, Sreenivasulu N, Weschke W, Stein N, Rudd S, Radchuk V, Potokina E, Scholz U, Schweizer P, Zierold U *et al*. 2004. Large-scale analysis of the barley transcriptome based on expressed sequence tags. *Plant J* **40:** (2) 276.

### 9 Functional genomics

Champy MF, Selloum M, Piard L, Zeitler V, Caradec C, Chambon P, Auwerx J. 2004. Mouse functional genomics requires standardization of mouse handling and housing conditions. *Mamm Genome* **15:** (10) 768.
Constantin GD, Krath BN, MacFarlane SA, Nicolaisen M, Johansen IE, Lund OS. 2004. Virus-induced gene silencing as a tool for functional genomics in a legume species. *Plant J* **40:** (4) 622.
Hope IA, Stevens J, Garner A, Hayes J, Cheo DL, Brasch MA, Vidal M. 2004. Feasibility of genome-scale construction of promoter::reporter gene fusions for expression in *Caenorhabditis elegans* using a Multi-Site Gateway recombination system. *Genome Res* **14:** (10B) 2070.
Sims AH, Gent ME, Robson GD, Dunn-Coleman NS, Oliver SG. 2004. Combining transcriptome data with genomic and cDNA sequence alignments to make confident functional assignments for *Aspergillus nidulans* genes. *Mycol Res* **108:** (8) 853.
Tsai JM, Wang HC, Leu JH, Hsiao HH, Wang AHJ, Kou GH, Lo CF. 2004. Genomic and proteomic analysis of thirty-nine structural proteins of shrimp white spot syndrome virus. *J Virol* **78:** (20) 11360.
Wiemann S, Arlt D, Huber W, Wellenreuther R, Schleeger S, Mehrle A, Bechtel S, Sauermann M, Korf U, Pepperkok R *et al*. 2004. From ORFeome to biology: A functional genomics pipeline. *Genome Res* **14:** (10B) 2136.

### 10 Transcriptomics

Allen TD, Nuss DL. 2004. Linkage between mitochondrial hypovirulence and viral hypovirulence in the chestnut blight fungus revealed by cDNA microarray analysis. *Eukaryot Cell* **3:** (5) 1227.
Armengaud P, Breitling R, Amtmann A. 2004. The potassium-dependent transcriptome of *Arabidopsis* reveals a prominent role of jasmonic acid in nutrient signaling. *Plant Physiol* **136:** (1) 2556.
Christopher ME, Miranda M, Major IT, Constabel CP. 2004. Gene expression profiling of systemically wound-induced defenses in hybrid poplar. *Planta* **219:** (6) 936.
Downie A, Miyazaki S, Bohnert H, John P, Coleman J, Parry M, Haslam R. 2004. Expression profiling of the response of *Arabidopsis thaliana* to methanol stimulation. *Phytochemistry* **65:** (16) 2305.

Copyright © 2005 John Wiley & Sons, Ltd. 

Gatti L, Chen D, Beretta GL, Rustici G, Carenini N, Corna E, Colangelo D, Zunino F, Bahler J, Perego P. 2004. Global gene expression of fission yeast in response to cisplatin. *Cell Mol Life Sci* **61:** (17) 2253.

Gibly A, Bonshtien A, Balaji V, Debbie P, Martin GB, Sessa G. 2004. Identification and expression profiling of tomato genes differentially regulated during a resistance response to *Xanthomonas campestris* pv. *vesicatoria*. *Mol Plant Microbe Interact* **17:** (11) 1212.

Harcus D, Nantel A, Marcil A, Rigby T, Whiteway M. 2004. Transcription profiling of cyclic AMP signaling in *Candida albicans*. *Mol Biol Cell* **15:** (10) 4490.

Karos M, Vilarino C, Bollschweiler C, Revuelta JL. 2004. A genome-wide transcription analysis of a fungal riboflavin overproducer. *J Biotechnol* **113:** (1-3) 69.

Lu J, Lal A, Merriman B, Nelson S, Riggins G. 2004. A comparison of gene expression profiles produced by SAGE, long SAGE, and oligonucleotide chips. *Genomics* **84:** (4) 631.

Marin K, Kanesaki Y, Los DA, Murata N, Suzuki I, Hagemann M. 2004. Gene expression profiling reflects physiological processes in salt acclimation of *Synechocystis* sp strain PCC 6803. *Plant Physiol* **136:** (2) 3290.

Philip-Couderc P, Smih F, Hall JE, Pathak A, Roncalli J, Harmancey R, Massabuau P, Galinier M, Verwaerde P, Senard JM *et al*. 2004. Kinetic analysis of cardiac transcriptome regulation during chronic high-fat diet in dogs. *Physiol Genomics* **19:** (1) 32.

Roos V, Andersson CIJ, Bulow L. 2004. Gene expression profiling of *Escherichia* coli expressing double *Vitreoscilla* haemoglobin. *J Biotechnol* **114:** (1-2) 107.

Schrader J, Moyle R, Bhalerao R, Hertzberg M, Lundeberg J, Nilsson P, Bhalerao RP. 2004. Cambial meristem dormancy in trees involves extensive remodelling of the transcriptome. *Plant J* **40:** (2) 173.

Stolc V, Gauhar Z, Mason C, Halasz G, Van Batenburg MF, Rifkin SA, Hua SJ, Herreman T, Tongprasit W, Barbano PE *et al*. 2004. A gene expression map for the euchromatic genome of *Drosophila melanogaster*. *Science* **306:** (5296) 655.

Stover T, Wissel K, Averbeck T, Lenarz T, Altschuler RA. 2004. Characterisation of the gene expression profiles in the inner ear and the colliculus inferior of normal and deafened rats by gene-array-technology (German, English Abstract). *Laryngorhinootologie* **83:** (9) 597.

Suganuma N, Yamamoto A, Itou A, Hakoyama T, Banba M, Hata S, Kawaguchi M, Kouchi H. 2004. cDNA macroarray analysis of gene expression in ineffective nodules induced on the *Lotus japonicus sen1* mutant. *Mol Plant Microbe Interact* **17:** (11) 1223.

Tanaka T, Horikawa Y, Kawamoto T, Kabe-Sakurai N, Takeda J, Mikuni M. 2004. Expression profile of mRNAs from rat hippocampus and its application to microarray. *Mol Brain Res* **129:** (1-2) 20.

Van Hemert S, Hoekman AJ, Smits MA, Rebel JMJ. 2004. Differences in intestinal gene expression profiles in broiler lines varying in susceptibility to malabsorption syndrome. *Poult Sci* **83:** (10) 1675.

Zhang ZD, Shrager J, Jain M, Chang DW, Vallon O, Grossman AR. 2004. Insights into the survival of *Chlamydomonas reinhardtii* during sulfur starvation based on microarray analysis of gene expression. *Eukaryot Cell* **3:** (5) 1331.

### II Proteomics

Bernardini G, Renzone G, Comanducci M, Mini R, Arena S, D'Ambrosio C, Bambini S, Trabalzini L, Grandi G, Martelli P *et al*. 2004. Proteome analysis of *Neisseria meningitidis* serogroup A. *Proteomics* **4:** (10) 2893.

Chan LL, Hodgkiss IJ, Wan JMF, Lum JHK, Mak ASC, Sit WH, Lo SCL. 2004. Proteomic study of a model causative agent of harmful algal blooms, *Prorocentrum triestinum* II: The use of differentially expressed protein profiles under different growth phases and growth conditions for bloom prediction. *Proteomics* **4:** (10) 3214.

Coughenour HD, Spaulding RS, Thompson CM. 2004. The synaptic vesicle proteome: A comparative study in membrane protein identification. *Proteomics* **4:** (10) 3141.

Eriksson J, Fenyo D. 2004. The statistical significance of protein identification results as a function of the number of protein sequences searched. *J Proteome Res* **3:** (5) 979.

Eymann C, Dreisbach A, Albrecht D, Bernhardt J, Becher D, Gentner S, Tam LT, Buttner K, Buurman G, Scharf C *et al*. 2004. A comprehensive proteome map of growing *Bacillus subtilis* cells. *Proteomics* **4:** (10) 2849.

Folio P, Chavant P, Chafsey I, Belkorchia A, Chambon C, Hebraud M. 2004. Two-dimensional electrophoresis database of *Listeria monocytogenes* EGDe proteome and proteomic analysis of mid-log and stationary growth phase cells. *Proteomics* **4:** (10) 3187.

Hettick JM, Kashon ML, Simpson JP, Siegel PD, Mazurek GH, Weissman DN. 2004. Proteomic profiling of intact mycobacteria by matrix-assisted laser desorption/ionization time-of-flight mass spectrometry. *Anal Chem* **76:** (19) 5769.

Ho EM, Chang HW, Kim SI, Kahng HY, Oh KH. 2004. Analysis of TNT (2,4,6-trinitrotoluene)-inducible cellular responses and stress shock proteome in *Stenotrophomonas* sp OK-5. *Curr Microbiol* **49:** (5) 346.

Kaneko M, Masui R, Ake K, Kousumi Y, Kuramitsu S, Yamaguchi M, Kuyama H, Ando E, Norioka S, Nakazawa T *et al*. 2004. Rapid and sensitive amino-acid sequencing of cloning *Thermus thermophilus* HB8 ferredoxin by proteomics. *J Proteome Res* **3:** (5) 983.

Kazemi-Pour N, Condemine G, Hugouvieux-Cotte-Pattat N. 2004. The secretome of the plant pathogenic bacterium *Erwinia chrysanthemi*. *Proteomics* **4:** (10) 3177.

Li N, Shaw ARE, Zhang N, Mak A, Li L. 2004. Lipid raft proteomics: Analysis of in-solution digest of sodium dodecyl sulfate-solubilized lipid raft proteins by liquid chromatography-matrix-assisted laser desorption/ionization tandem mass spectrometry. *Proteomics* **4:** (10) 3156.

Liska AJ, Shevchenko A, Pick U, Katz A. 2004. Enhanced photosynthesis and redox energy production contribute to salinity tolerance in *Dunaliella* as revealed by homology-based proteomics. *Plant Physiol* **136:** (1) 2806.

Lu H, Li WQ, Noble WS, Payan D, Anderson DC. 2004. Riboproteomics of the hepatitis C virus internal ribosomal entry site. *J Proteome Res* **3:** (5) 949.

Marques MAM, Espinosa BJ, Da Silveira EKX, Pessolani MCV, Chapeaurouge A, Perales J, Dobos KM, Belisle JT, Spencer JS, Brennan PJ. 2004. Continued proteomic analysis of *Mycobacterium leprae* subcellular fractions. *Proteomics* **4:** (10) 2942.

Maynard DM, Masuda J, Yang XY, Kowalak JA, Markey SP. 2004. Characterizing complex peptide mixtures using a multi-dimensional liquid chromatography-mass spectrometry system: *Saccharomyces cerevisiae* as a model system. *J Chromatogr B* **810:** (1) 69.

Miller I, Friedlein A, Tsangaris G, Maris A, Fountoulakis M, Gemeiner M. 2004. The serum proteome of *Equus caballus*. *Proteomics* **4:** (10) 3227.

Newton KA, Pitteri SJ, Laskowski M, McLuckey SA. 2004. Effects of single amino acid substitution on the collision-induced dissociation of intact protein ions: Turkey ovomucoid third domain. *J Proteome Res* **3:** (5) 1033.

Okkels LM, Muller EC, Schmid M, Rosenkrands I, Kaufmann SHE, Andersen P, Jungblut PR. 2004. CFP10 discriminates between nonacetylated and acetylated ESAT-6 of *Mycobacterium tuberculosis* by differential interaction. *Proteomics* **4:** (10) 2954.

Panse VG, Hardeland U, Werner T, Kuster B, Hurt E. 2004. A proteome-wide approach identifies sumoylated substrate proteins in yeast. *J Biol Chem* **279:** (40) 41346.

Pleissner KP, Schmelzer P, Wehrl W, Jungblut PR. 2004. Presentation of differentially regulated proteins within a web-accessible proteome database system of microorganisms. *Proteomics* **4:** (10) 2987.

Rhomberg T, Karlberg O, Mini T, Zimny-Arndt U, Wickenberg U, Rottgen M, Jungblut PR, Jeno P, Andersson S, Dehio C. 2004. Proteomic analysis of the sarcosine-insoluble outer membrane fraction of the bacterial pathogen *Bartonella henselae*. *Proteomics* **4:** (10) 3021.

Rosen R, Becher D, Buttner K, Biran D, Hecker M, Ron EZ. 2004. Highly phosphorylated bacterial proteins. *Proteomics* **4:** (10) 3068.

Sam-Yellowe TY, Florens L, Wang TM, Raine JD, Carucci DJ, Sinden R, Yates JR. 2004. Proteome analysis of rhoptry-enriched fractions isolated from *Plasmodium* merozoites. *J Proteome Res* **3:** (5) 995.

Schaumburg J, Diekmann O, Hagendorff P, Bergmann S, Rohde M, Hammerschmidt S, Jansch L, Wehland J, Karst U. 2004. The cell wall subproteome of *Listeria monocytogenes*. *Proteomics* **4:** (10) 2991.

Selbach M, Moese S, Backert S, Jungblut PR, Meyer TF. 2004. The *Helicobacter pylori* CagA protein induces tyrosine dephosphorylation of ezrin. *Proteomics* **4:** (10) 2961.

Copyright © 2005 John Wiley & Sons, Ltd. 

Strader MB, VerBerkmoes NC, Tabb DL, Connelly HM, Barton JW, Bruce BD, Pelletier DA, Davison BH, Hettich RL, Larimer FW *et al*. 2004. Characterization of the 70S ribosome from *Rhodopseudomonas palustris* using an integrated "top-down" and "bottom-up" mass spectrometric approach. *J Proteome Res* **3:** (5) 965.

Tsai IH, Chen YH, Wang YM. 2004. Comparative proteomics and subtyping of venom phospholipases $A_2$ and disintegrins of *Protobothrops* pit vipers. *Biochim Biophys Acta* **1702:** (1) 111.

Warscheid B, Fenselau C. 2004. A targeted proteomics approach to the rapid identification of bacterial cell mixtures by matrix-assisted laser desorption/ionization mass spectrometry. *Proteomics* **4:** (10) 2877.

Wilmarth PA, Riviere MA, Rustvold DL, Lauten JD, Madden TE, David LL. 2004. Two-dimensional liquid chromatography study of the human whole saliva proteome. *J Proteome Res* **3:** (5) 1017.

Ziebandt AK, Becher D, Ohlsen K, Hacker J, Hecker M, Engelmann S. 2004. The influence of *agr* and $\sigma^B$ in growth phase dependent regulation of virulence factors in *Staphylococcus aureus*. *Proteomics* **4:** (10) 3034.

### I2 Protein structural genomics

Bray J, Marsden RL, Rison SCG, Savchenko A, Edwards AM, Thornton JM, Orengo CA. 2004. A practical and robust sequence search strategy for structural genomics target selection. *Bioinformatics* **20:** (14) 2288.

Chance MR, Fiser A, Sali A, Pieper U, Eswar N, Xu GP, Fajardo JE, Radhakannan T, Marinkovic N. 2004. High-throughput computational and experimental techniques in structural genomics. *Genome Res* **14:** (10B) 2145.

Chou KC. 2004. Insights from modeling the tertiary structure of human BACE2. *J Proteome Res* **3:** (5) 1069.

Dolinsky TJ, Burgers PMJ, Karplus K, Baker NA. 2004. SPrCY: Comparison of structural predictions in the *Saccharomyces cerevisiae* genome. *Bioinformatics* **20:** (14) 2312.

Von Ohsen N, Sommer I, Lengauer T. 2004. Arby: Automatic protein structure prediction using profile-profile alignment and confidence measures. *Bioinformatics* **20:** (14) 2228.

### I3 Metabolomics

Franceschetti M, Perry B, Thompson B, Hanfrey C, Michael AJ. 2004. Expression proteomics identifies biochemical adaptations and defense responses in transgenic plants with perturbed polyamine metabolism. *FEBS Lett* **576:** (3) 477.

Oh JE, Krapfenbauer K, Lubec G. 2004. Proteomic basis for the possible use of lymphocytes for metabolic screenings. *Amino Acids* **27:** (2) 141.

Osiro D, Muniz JRC, Della Coleta H, De Sousa AA, Machado MA, Garratt RC, Colnago LA. 2004. Fatty acid synthesis in *Xylella fastidiosa*: Correlations between genome studies, $^{13}$C NMR data, and molecular models. *Biochem Biophys Res Commun* **323:** (3) 987

Ruf F, Sealfon SC. 2004. Genomics view of gonadotrope signaling circuits. *Trends Endocrinol Metab* **15:** (7) 331.

Scheible WR, Morcuende R, Czechowski T, Fritz C, Osuna D, Palacios-Rojas N, Schindelasch D, Thimm O, Udvardi MK, Stitt M. 2004. Genome-wide reprogramming of primary and secondary metabolism, protein synthesis, cellular growth processes, and the regulatory infrastructure of *Arabidopsis* in response to nitrogen. *Plant Physiol* **136:** (1) 2483.

Smith SM, Fulton DC, Chia T, Thorneycroft D, Chapple A, Dunstan H, Hylton C, Zeeman SC, Smith AM. 2004. Diurnal changes in the transcriptome encoding enzymes of starch metabolism provide evidence for both transcriptional and posttranscriptional regulation of starch metabolism in *Arabidopsis* leaves. *Plant Physiol* **136:** (1) 2687.

Wang RC, Tischner R, Gutierrez RA, Hoffman M, Xing XJ, Chen MS, Coruzzi G, Crawford NM. 2004. Genomic analysis of the nitrate response using a nitrate reductase-null mutant of *Arabidopsis*. *Plant Physiol* **136:** (1) 2512.

### I4 Genomic approaches to development

Fujishima N, Hirokawa M, Aiba N, Ichikawa Y, Fujishima M, Komatsuda A, Suzuki Y, Kawabata Y, Miura I, Sawada K. 2004. Gene expression profiling of human erythroid progenitors by micro-serial analysis of gene expression. *Int J Hematol* **80:** (3) 239.

Puente LG, Carriere JF, Kelly JF, Megeney LA. 2004. Comparative analysis of phosphoprotein-enriched myocyte proteomes reveals widespread alterations during differentiation. *FEBS Lett* **574:** (1-3) 138.

Wang DJ, Park JS, Chu JSF, Krakowski A, Luo KX, Chen DJ, Li S. 2004. Proteomic profiling of bone marrow mesenchymal stem cells upon transforming growth factor $\beta$1 stimulation. *J Biol Chem* **279:** (42) 43725.

Whitworth K, Springer GK, Forrester LJ, Spollen WG, Ries J, Lamberson WR, Bivens N, Murphy CN, Mathialigan N, Green JA *et al*. 2004. Developmental expression of 2489 gene clusters during pig embryogenesis: An expressed sequence tag project. *Biol Reprod* **71:** (4) 1230.

Yu DH, Lee KH, Lee JY, Kim S, Shin DM, Kim JH, Lee YS, Lee YS, Oh SK, Moon SY *et al*. 2004. Changes of gene expression profiles during neuronal differentiation of central nervous system precursors treated with ascorbic acid. *J Neurosci Res* **78:** (1) 29.

Yu JD, He S, Friedman JS, Akimoto M, Ghosh D, Mears AJ, Hicks D, Swaroop A. 2004. Altered expression of genes of the Bmp/Smad and Wnt/calcium signaling pathways in the cone-only Nrl$^{-/-}$ mouse retina, revealed by gene profiling using custom cDNA microarrays. *J Biol Chem* **279:** (40) 42211.

### I5 Technological advances

Belosludtsev YY, Bowerman D, Weil R, Marthandan N, Balog R, Luebke K, Lawson J, Johnston SA, Lyons CR, O'Brien K *et al*. 2004. Organism identification using a genome sequence-independent universal microarray probe set. *Biotechniques* **37:** (4) 654.

Blackman RK, Amidon BS. 2004. Microarray approach for cloned library quality assessment and the comprehensive identification of positives from library screens. *Biotechniques* **37:** (4) 536.

Budnik BA, Moyer SC, Pittman JL, Ivleva VB, Sommer U, Costello CE, O'Connor PB. 2004. High pressure MALDI-FTMS: Implications for proteomics. *Int J Mass Spectrom* **234:** (1-3) 203.

Coleman MA, Lao VH, Segelke BW, Beernink PT. 2004. High-throughput, fluorescence-based screening for soluble protein expression. *J Proteome Res* **3:** (5) 1024.

De Figueiredo P, Roberts RL, Nester EW. 2004. DARTs: A DNA-based *in vitro* polypeptide display technology. *Proteomics* **4:** (10) 3128.

Dimalanta ET, Lim A, Runnheim R, Lamers C, Churas C, Forrest DK, De Pablo JJ, Graham MD, Coppersmith SN, Goldstein S *et al*. 2004. A microfluidic system for large DNA molecule arrays. *Anal Chem* **76:** (18) 5293.

Dumur CI, Nasim S, Best IM, Archer KJ, Ladd AC, Mas VR, Wilkinson DS, Garrett CT, Ferreira-Gonzales A. 2004. Evaluation of quality-control criteria for microarray gene expression analysis. *Clin Chem* **50:** (11) 1994.

Finnskog D, Ressine A, Laurell T, Marko-Varga G. 2004. Integrated protein microchip assay with dual fluorescent- and MALDI read-out. *J Proteome Res* **3:** (5) 988.

Fung KYC, Askovic S, Basile F, Duncan MW. 2004. A simple and inexpensive approach to interfacing high-performance liquid chromatography and matrix-assisted laser desorption/ionization-time of flight-mass spectrometry. *Proteomics* **4:** (10) 3121.

Hirschberg D, Jagerbrink T, Samskog J, Gustafsson M, Stahlberg M, Alvelius G, Husman B, Carlquist M, Jornvall H, Bergman T. 2004. Detection of phosphorylated peptides in proteomic analyses using microfluidic compact disk technology. *Anal Chem* **76:** (19) 5864.

Jacquot JP, Rouhier N, Navrot N, Decottignies JP. 2004. Generation of polyclonal antibodies against multiple proteins in a single rabbit and subsequent isolation of the specific immunoglobulins as a tool for proteomics research. *Biotechnol Appl Biochem* **40:** (2) 181.

Kaposi-Novak P, Lee JS, Mikaelyan A, Patel V, Thorgeirsson SS. 2004. Oligonucleotide microarray analysis of aminoallyl-labeled cDNA targets from linear RNA amplification. *Biotechniques* **37:** (4) 580.

Krah A, Wessel R, Pleissner KP. 2004. Assessment of protein spot components applying correspondence analysis for peptide mass fingerprint data. *Proteomics* **4:** (10) 2982.

Lee YH, Han H, Chang SB, Lee SW. 2004. Isotope-coded N-terminal sulfonation of peptides allows quantitative proteomic analysis with in-

Copyright © 2005 John Wiley & Sons, Ltd. 

creased *de novo* peptide sequencing capability. *Rapid Commun Mass Spectrom* **18:** (24) 3019.
Liu T, Qian WJ, Strittmatter EF, Camp DG, Anderson GA, Thrall BD, Smith RD. 2004. High-throughput comparative proteome analysis using a quantitative cysteinyl-peptide enrichment technology. *Anal Chem* **76:** (18) 5345.
Mattow J, Schmidt F, Hohenwarter W, Siejak F, Schaible UE, Kaufmann SHE. 2004. Protein identification and tracking in two-dimensional electrophoretic gels by minimal protein identifiers. *Proteomics* **4:** (10) 2927.
Metsis A, Andersson U, Bauren G, Ernfors P, Lonnerberg P, Montelius A, Oldin M, Pihlak A, Linnarsson S. 2004. Whole-genome expression profiling through fragment display and combinatorial gene identification - Online article no. e127. *Nucleic Acids Res* **32:** (16) e127.
Olsen JV, Mann M. 2004. Improved peptide identification in proteomics by two consecutive stages of mass spectrometric fragmentation. *Proc Natl Acad Sci U S A* **101:** (37) 13417.
Prinz T, Muller J, Kuhn K, Schafer J, Thompson A, Schwarz J, Hamon C. 2004. Characterization of low abundant membrane proteins using the protein sequence tag technology. *J Proteome Res* **3:** (5) 1073.
Qin LX, Kerr KF. 2004. Empirical evaluation of data transformations and ranking statistics for microarray analysis. *Nucleic Acids Res* **32:** (18) 5471.
Rozing G, Nagele E, Horth P, Vollmer M, Moritz R, Glatz B, Gratzfeld-Husgen A. 2004. Instrumentation for advanced micro separations in pharmaceutical analysis and proteomics. *J Biochem Biophys Methods* **60:** (3) 233.
Sioud M, Rosok O. 2004. Profiling microRNA expression using sensitive cDNA probes and filter arrays. *Biotechniques* **37:** (4) 574.
Suzuki Y, Kawazu T, Koyama H. 2004. RNA isolation from siliques, dry seeds, and other tissues of *Arabidopsis thaliana*. *Biotechniques* **37:** (4) 542.
Tian H, Cao LC, Tan YP, Williams S, Chen LL, Matray T, Chenna A, Moore S, Hernandez V, Xiao V *et al*. 2004. Multiplex mRNA assay using electrophoretic tags for high-throughput gene expression analysis - Online article no. e126. *Nucleic Acids Res* **32:** (16) e126.
Vocadlo DJ, Bertozzi CR. 2004. A strategy for functional proteomic analysis of glycosidase activity from cell lysates. *Angew Chem Int Ed* **43:** (40) 5338.
Wang J, Robinson JF, Khan HMR, Carter DE, McKinney J, Miskie BA, Hegele RA. 2004. Optimizing RNA extraction yield from whole blood for microarray gene expression analysis. *Clin Biochem* **37:** (9) 741.
White IR, Pickford R, Wood J, Skehel JM, Gangadharan B, Cutler P. 2004. A statistical comparison of silver and SYPRO Ruby staining for proteomic analysis. *Electrophoresis* **25:** (17) 3048.
Yu YL, Cui JF, Wang XY, Liu YK, Yang PY. 2004. Studies on peptide acetylation for stable-isotope labeling after 1-D PAGE separation in quantitative proteomics. *Proteomics* **4:** (10) 3112.
Zhang D, Baek SH, Ho A, Lee H, Jeong YS, Kim K. 2004. Targeted degradation of proteins by small molecules: A novel tool for functional proteomics. *Comb Chem High Throughput Scr* **7:** (7) 689.
Zhou XC, Zhou JZ. 2004. Improving the signal sensitivity and photostability of DNA hybridizations on microarrays by using dye-doped core-shell silica nanoparticles. *Anal Chem* **76:** (18) 5302.

### I6 Bioinformatics

Cargile BJ, Bundy JL, Stephenson JL. 2004. Potential for false positive identifications from large databases through tandem mass spectrometry (Letter). *J Proteome Res* **3:** (5) 1082.
Carter SL, Brechbuhler CM, Griffin M, Bond AT. 2004. Gene co-expression network topology provides a framework for molecular characterization of cellular state. *Bioinformatics* **20:** (14) 2242.
Chen NS, Lawson D, Bradnam K, Harris TW, Stein LD. 2004. WormBase as an integrated platform for the *C. elegans* ORFeome. *Genome Res* **14:** (10B) 2155.
Ciria R, Abreu-Goodger C, Morett E, Merino E. 2004. GeConT: Gene context analysis. *Bioinformatics* **20:** (14) 2307.
Egelund J, Skjot M, Geshi N, Ulvskov P, Petersen BL. 2004. A complementary bioinformatics approach to identify potential plant cell wall glycosyltransferase-encoding genes. *Plant Physiol* **136:** (1) 2609.
Ekstrom CT, Bak S, Kristensen C, Rudemo M. 2004. Spot shape modelling and data transformations for microarrays. *Bioinformatics* **20:** (14) 2270.
Endo TA. 2004. Probabilistic nucleotide assembling method for sequencing by hybridization. *Bioinformatics* **20:** (14) 2181.
Geer LY, Markey SP, Kowalak JA, Wagner L, Xu M, Maynard DM, Yang XY, Shi WY, Bryant SH. 2004. Open mass spectrometry search algorithm. *J Proteome Res* **3:** (5) 958.
Gordon PMK, Sensen CW. 2004. Osprey: A comprehensive tool employing novel methods for the design of oligonucleotides for DNA sequencing and microarrays - Online article no. e133. *Nucleic Acids Res* **32:** (17) e133.
Heredia-Langner A, Cannon WR, Jarman KD, Jarman KH. 2004. Sequence optimization as an alternative to *de novo* analysis of tandem mass spectrometry data. *Bioinformatics* **20:** (14) 2296.
Huber T, Faulkner G, Hugenholtz P. 2004. Bellerophon: A program to detect chimeric sequences in multiple sequence alignments. *Bioinformatics* **20:** (14) 2317.
Iragne F, Barre A, Goffard N, De Daruvar A. 2004. AliasServer: A web server to handle multiple aliases used to refer to proteins. *Bioinformatics* **20:** (14) 2331.
Kantardjieff KA, Rupp B. 2004. Protein isoelectric point as a predictor for increased crystallization screening efficiency. *Bioinformatics* **20:** (14) 2162.
Katayama S, Kanamori M, Hayashizaki Y. 2004. Integrated analysis of the genome and the transcriptome by FANTOM. *Brief Bioinform* **5:** (3) 249.
Le Meur N, Lamirault G, Bihouee A, Steenman M, Bedrine-Ferran H, Teusan R, Ramstein G, Leger JJ. 2004. A dynamic, web-accessible resource to process raw microarray scan data into consolidated gene expression values: Importance of replication. *Nucleic Acids Res* **32:** (18) 5349.
Lu HC, Zhu XP, Liu HF, Skogerbo G, Zhang JF, Zhang Y, Cai L, Zhao Y, Sun SW, Xu JY, Bu DB, Chen R. 2004. The interactome as a tree - An attempt to visualize the protein-protein interaction network in yeast. *Nucleic Acids Res* **32:** (16) 4804.
Pop M, Phillippy A, Delcher AL, Salzberg SL. 2004. Comparative genome assembly. *Brief Bioinform* **5:** (3) 237.
Qian B, Goldstein RA. 2004. Performance of an iterated T-HMM for homology detection. *Bioinformatics* **20:** (14) 2175.
Ringner M, Veerla S, Andersson S, Staaf J, Hakkinen J. 2004. ACID: A database for microarray clone information. *Bioinformatics* **20:** (14) 2305.
Robinson SJ, Cram DJ, Lewis CT, Parkin IAP. 2004. Maximizing the efficacy of SAGE analysis identifies novel transcripts in *Arabidopsis*. *Plant Physiol* **136:** (2) 3223.
Robson B, Mushlin R. 2004. Genomic messaging system and DNA mark-up language for information-based personalized medicine with clinical and proteome research applications. *J Proteome Res* **3:** (5) 930.
Ruepp A, Zollner A, Maier D, Albermann K, Hani J, Mokrejs M, Tetko I, Guldener U, Mannhaupt G, Munsterkotter M *et al*. 2004. The FunCat, a functional annotation scheme for systematic classification of proteins from whole genomes. *Nucleic Acids Res* **32:** (18) 5539.
Supek F, Vlahovicek K. 2004. INCA: Synonymous codon usage analysis and clustering by means of self-organizing map. *Bioinformatics* **20:** (14) 2329.
Susko E, Roger AJ. 2004. Estimating and comparing the rates of gene discovery and expressed sequence tag (EST) frequencies in EST surveys. *Bioinformatics* **20:** (14) 2279.
Tsai YT, Huang YP, Yu CT, Lu CL. 2004. MuSiC: A tool for multiple sequence alignment with constraints. *Bioinformatics* **20:** (14) 2309.
Yang XY, Dondeti V, Dezube R, Maynard DM, Geer LY, Epstein J, Chen XF, Markey SP, Kowalak JA. 2004. DBParser: Web-based software for shotgun proteomic data analyses. *J Proteome Res* **3:** (5) 1002.
Zimmermann P, Hirsch-Hoffmann M, Hennig L, Gruissem W. 2004. GENEVESTIGATOR. *Arabidopsis* microarray database and analysis toolbox. *Plant Physiol* **136:** (1) 2621.

Copyright © 2005 John Wiley & Sons, Ltd.
